# Animal Models for Tuberculosis in Translational and Precision Medicine

**DOI:** 10.3389/fmicb.2017.00717

**Published:** 2017-05-04

**Authors:** Lingjun Zhan, Jun Tang, Mengmeng Sun, Chuan Qin

**Affiliations:** ^1^Key Laboratory of Human Disease Comparative Medicine, Ministry of HealthBeijing, China; ^2^Institution of Laboratory Animal Sciences, Centre for Tuberculosis, Chinese Academy of Medical Sciences and Peking Union Medical CollegeBeijing, China; ^3^Beijing Key Laboratory for Animal Models of Emerging and Reemerging InfectiousBeijing, China; ^4^Beijing Engineering Research Center for Experimental Animal Models of Human Critical DiseasesBeijing, China; ^5^Key Laboratory of Human Diseases Animal Model, State Administration of Traditional Chinese MedicineBeijing, China

**Keywords:** tuberculosis, animal models, comparative medicine, translational medicine, precision medicine

## Abstract

Tuberculosis (TB) is a health threat to the global population. Anti-TB drugs and vaccines are key approaches for TB prevention and control. TB animal models are basic tools for developing biomarkers of diagnosis, drugs for therapy, vaccines for prevention and researching pathogenic mechanisms for identification of targets; thus, they serve as the cornerstone of comparative medicine, translational medicine, and precision medicine. In this review, we discuss the current use of TB animal models and their problems, as well as offering perspectives on the future of these models.

## Introduction

The number of tuberculosis (TB) patients has been increasing, and in recent years TB became the leading cause of death around the world. In 2015, 10.4 million people were diagnosed with TB, of which 480,000 cases were drug-resistant according to the WHO TB report. Anti-TB drugs, vaccines, and diagnostic reagents are essential tools for TB prevention and control, TB animal models have been widely used for development of these tools, as well as for research on pathogenic mechanisms of TB, constituting 35% TB-related research found in the NCBI database. Animal models of TB play a key role in translational medicine, basic medicine, and biology of TB (Moskovic et al., 1993; Coquard et al., 1997; El Husseiny et al., 2002; Naaman et al., 2011; Durnali et al., 2012).

In this paper, we review the current use of TB animal models in medical research, as well as problems, progress, and perspectives associated with them.

## Current use of TB animal models

**Figure 1:**
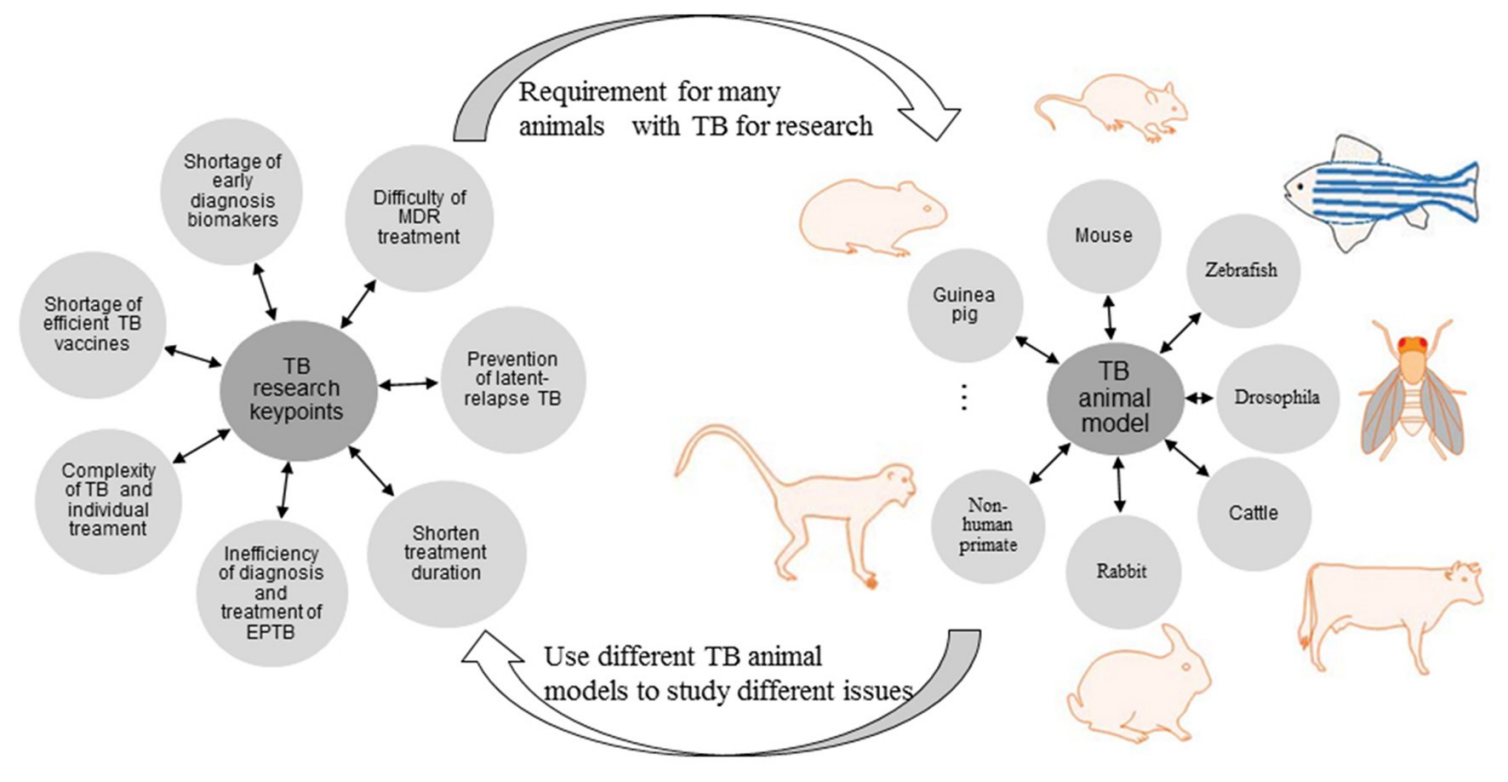
**Current problems in diagnosis, treatment, and prevention of TB and the role of TB animal models**.

Many animal species have been used for TB models, with mice, guinea pigs, rabbits, and non-human primates among the most commonly used. Each model mimics one or more features of human TB, including clinical signs, pathological changes, bacteria loads in organs, disease progressions or immunological parameters (Rodgers et al., 2007; Waters et al., 2011; Clark et al., 2015; Myllymaki et al., 2015; Peng et al., 2015; Provan and Newland, 2015; Kramnik and Beamer, 2016; Phuah et al., 2016). Characteristics, as well as pros and cons, of current TB animal models are summarized, the utility of each model according to the pros and cons was also speculated. For instance, mouse active TB model is suitable for rapid anti-TB chemical drugs evaluation, due to the homogeneous pathological change and bacteria burden; guinea pig presents sensitive immune response when infection, thus it is a good model for anti-TB vaccine evaluation; monkey TB model has similar clinical signs and classic granulomas structure to that of patient, therefore it is priority for mechanisms of disease research. See in Table 1 and Figure 1.

**Table 1:** **Characteristics and utilization of TB animal models**.

**Animal strains**	**Strain selection**	**Infection method**	**Model type**	**Pros in medical research**	**Cons in medical research**	**Application**
C57BL/6 BALB/c C3HeB/FeJ mice	H37Rv Multi-drug resistant Mycobacterium tuberculosis (MDR) (10^5^–10^7^CFU)	Tail vein, aerosol	Active TB	Small size, easy to operate, low cost Have clear genetic background, abundant immune reagent, immune mechanism of TB can be studied in mice (Nicolle et al., 2004; Calderon et al., 2013; Commandeur et al., 2014) The role of certain genes or proteins in tuberculosis mechanisms could be investigated in genetically engineered mice (Calderon et al., 2013; Olleros et al., 2015) Humanized mouse TB model could be a candidate model of HIV and TB co-infection (Nusbaum et al., 2016)	No obvious clinical manifestation of tuberculosis infection (Lanoix et al., 2015; Lanoix, 2016) The TB granuloma structure is different to that of humans, without Langhans giant cells and class epithelioid cells in peripheral granulomas, do not form necrotic lesions in granuloma except in C3HeB/FeJ mice strain (Lanoix et al., 2015; Lanoix, 2016) No disseminated disease throughout the whole body (Shi et al., 2011) Inter-individual variation in infection outcome. Both pathological lesions and bacterial loads in organs were non-uniform (Scanga et al., 1999; Botha and Ryffel, 2002; Jacobs et al., 2015)	Research on mechanisms of TB immunological response Role of a specific gene in TB Rapid evaluation of anti-tuberculosis drugs and vaccines (Gouveia et al., 2013; Izzo et al., 2015)
C57BL/6 C3HeB/FeJ mice	H37Rv 10^2^–10^3^ CFU	Tail vein, aerosol	Latent TB infection (LTBI)	Spontaneous LTBI model could be obtained (Zhan et al., 2015) Modified LTBI model could also be obtained after drug or vaccine intervention (Lenaerts et al., 2004; Nuermberger et al., 2004) Mild granulomas lesions appeared in lung, spleen, and liver. Bacteria burdens kept low level throughout the latency phase, then relapsed with aggravated lesion and higher bacteria load levels (Zhang et al., 2009)	The latency length and relapse level of TB show great variation within group, and the latent-relapse period very long (Myllymaki et al., 2015) Tissue bacterial loads were at high level at latency phase Lack of predictors for recurrence (Shi et al., 2011)	Research on mechanisms of latency and relapse Research on prevention and control of the incubation period, including development of drugs and vaccines
Hartz guinea pig	H37Rv 10^3^–10^5^ CFU	Aerosol, subcutaneous	Active TB, several kinds of vaccine evaluation	Very susceptible to TB Miliary nodules observed in lung, liver, and spleen; tuberculous granuloma very similar to that in humans, with caseous necrosis (Kashino et al., 2008; Clark et al., 2015) Anti-TB drugs and vaccination have a good response (Clark et al., 2015)	Lack of specific immune reagents, so more difficult to research underlying mechanisms (Clark et al., 2015) Lack of general clinical manifestations of TB (Kashino et al., 2008; Clark et al., 2015) Cannot spontaneously develop latent infection (Kashino et al., 2008)	Drug evaluation Evaluation the safty and efficacy of vaccines or immunity strategies, such as primary immunity, prime-boost, and therapeutic vaccines pathological response of host, Mtb coevolution *in vivo*
The New Zealand rabbit	H37Rv 10^8^ CFU	Spinal punching, aerosol	Pulmonary, bone, meningeal, and cutaneous TB (Manabe et al., 2008; Dannenberg, 2009; Sun et al., 2012; Peng et al., 2015; Rahyussalim et al., 2016)	TB granulomas with necrosis and liquefaction, and easy to form cavitation Rabbit spinal tuberculosis was the best model to research treatment of bone tuberculosis Model for rarer forms of TB (cutaneous and meningeal	Clinical symptoms of TB not obvious Lack of relevant immune reagent Less susceptible to TB than guinea pigs, although slightly higher than mice	Preferred model for research on diagnosis and treatment of cavitary, spinal, and joint TB Good model for TB transmission research Could also be used for diagnosis and research of meningeal and cutaneous TB
Cynomolgus macaque/Rhesus monkey	H37Rv, Erdman, MDR 100–500 CFU	Via bronchoscope	LTBI and active TB (Scanga and Flynn, 2014; Izzo et al., 2015; Pena and Ho, 2015; Phuah et al., 2016)	Can mimic a variety of clinical manifestations, such as low fever, emaciation, cough, depressed, and dyspnea Can mimic LTBI and various forms of active TB progression Can develop pulmonary TB as well as extra-pulmonary in the liver, spleen, kidney, mediastinum, and occasionally the cerebellum and bone. Granuloma structure similar to humans, with classic Langerhans giant cells	Transgenic monkeys difficult to obtain, and limited availability of immune reagent, restricting the study of specific genes in TB High cost and space requirements limit the number of animals that can be used High variation within groups, making it difficult to evaluate the effectiveness of drugs and vaccines	Evaluation of individualized anti-tuberculosis drugs and vaccines, treatment strategy Study of the personalized mechanisms of disease, including pathological and immological response of host, Mtb coevolution *in vivo* (precision medecine)
Chinese tree shrew	H37Rv 10^3^–10^6^ CFU	Caudal vein, Inguinal vein	Active TB, TB-related pleural effusion (Zhan et al., 2014)	Have weight loss, low fever, reduced mobility Visible TB nodules in peritoneum, lung, kidneys, region along spine, and intercostal space Cutaneous lesions and pleural effusion common; cerebellar TB can be established Proteomics, and transcriptomics can be used to study the mechanism of TB	Clinical manifestation not obvious Granuloma structure different from human in lacking Langhans cells and caseous necrosis The same infection dose leads to different degrees of pathological changes The whole genome has been sequenced but not annotated Lack of immunological reagents.	Research on TB treatment against pulmonary, pleural effusion, cutaneous tuberculosis models Research on pathogenic mechanisms of Mtb
Wistar rat	HN878/W4 500 CFU	Tracheotomy	LTBI and active TB (Singhal et al., 2011a,b)	Lower cost, compared to larger animals Continuous latent infections facilitate study of biological characteristics of TB bacterium from early to late dormant phase Formation of pulmonary granuloma containing lymphocytes, macrophages, and PMN TB model could be obtained in genetic engineering rat	Relatively high pulmonary bacterial load in latent infection Granuloma structure dissimilar to that of human TB Rat are more resistant to Mtb infection than mice	Research on anti-TB drug absorption, distribution, metabolism, toxicology, and efficacy of drug TB-related gene and protein would be investigated in genetic engineering rat.
Castrated male Friesian-cross calf	*Mycobacterium bovis* 10^4^ CFU, *Mycobacterium avium* chester	Aerosol	Bovine TB	Pathological lesions observed around the lower respiratory tract, the upper, middle, and lower lung lobes, and mediastinal lymph nodes. Mtb culture also positive (Rodgers et al., 2007; Plattner et al., 2009) Good model for research on TB susceptibility genes (Driscoll et al., 2011) Fetal bovine TB model good for immune response research; difference in γδT cells in early granuloma formation led to the different anti-tuberculosis response in host (Plattner et al., 2009)	Have both intact and interrupt structural granulomas, both of which are different to that of human The ruptured granulomas is different to that of humans. In early phase, macrophages, lymphocytes, and a small amount of plasma cells make up the loose granuloma; in late phase, the scattered granulomas were encircled with epithelial cells but not fibroepithelial cells, and with necrosisor mineral deposits in the center,the majority of cells were WC1 (Baldwin and Telfer, 2015) The intact structural granulomas consisted of epithelial cell around granulomas and necrosis or cavitation in the center. Intact granulomas were comprised of γδT cells, Langerhans giant cells scattered in local lesions.(Plattner et al., 2009; Baldwin and Telfer, 2015)	Investigate the protective effects of vaccines (Vordermeier et al., 2001) Research immune response mechanism of TB (Plattner et al., 2009; Driscoll et al., 2011; Waters et al., 2011; Baldwin and Telfer, 2015) Good model for research on TB susceptibility genes
Zebrafish larvae and adult	*Mycobacterium marinum*	Local injection, Caudal vein injection	Latent infection (Parikka et al., 2012; van Leeuwen et al., 2015; Kiran et al., 2016)	Small size requires less space; fast breeding makes it easy to access; transparent body makes it easy to observe the interaction between bacteria and host. Larvae can be a particularly good model for TB innate immunity, while adults can be used for research on both innate and acquired immunity *Mycobacterium marinum* has no biological safety risk for researchers, with shorter replication cycles (4 h) and shorter research periods Research on the dynamics of granuloma formation, granuloma could form early at the first week post-infection Stable LTBI, chronic, and active infection models could all be obtained by controlling the bacterial infection dosage Adult and larval zebrafish models complement one another in studying disease mechanisms, certain genetic mutations lead to opposite phenotypes in juvenile and adult zebrafish	Could not be infected with standard strain H37Rv, but with *Mycobacterium marinum* Although the amino acid homology of *Mycobacterium tuberculosis* and *M. marinum* is close to 85%, the intracellular survival mechanisms are different and could not be reciprocally replaced Lack of zebrafish immunological reagents and specific antibodies, so mechanistic research on immune molecules and cells is difficult to carry out. Transcriptome and sequencing is the only method to study the immune response. Lack of clinical symptoms and manifestation of TB; therefore, unable to mimic most forms of human TB	Best model for bacterial virulence studies Evaluation of efficacy and toxicity of anti-tuberculosis compounds Good model for host susceptibility research Research on dynamics of granuloma formation Larvae can be a particularly good model for TB innate immunity, while adults can be used for research on both innate and acquired immunity

## Current problems in diagnosis, treatment, and prevention of TB and the role of animal models

**Figure 2:**
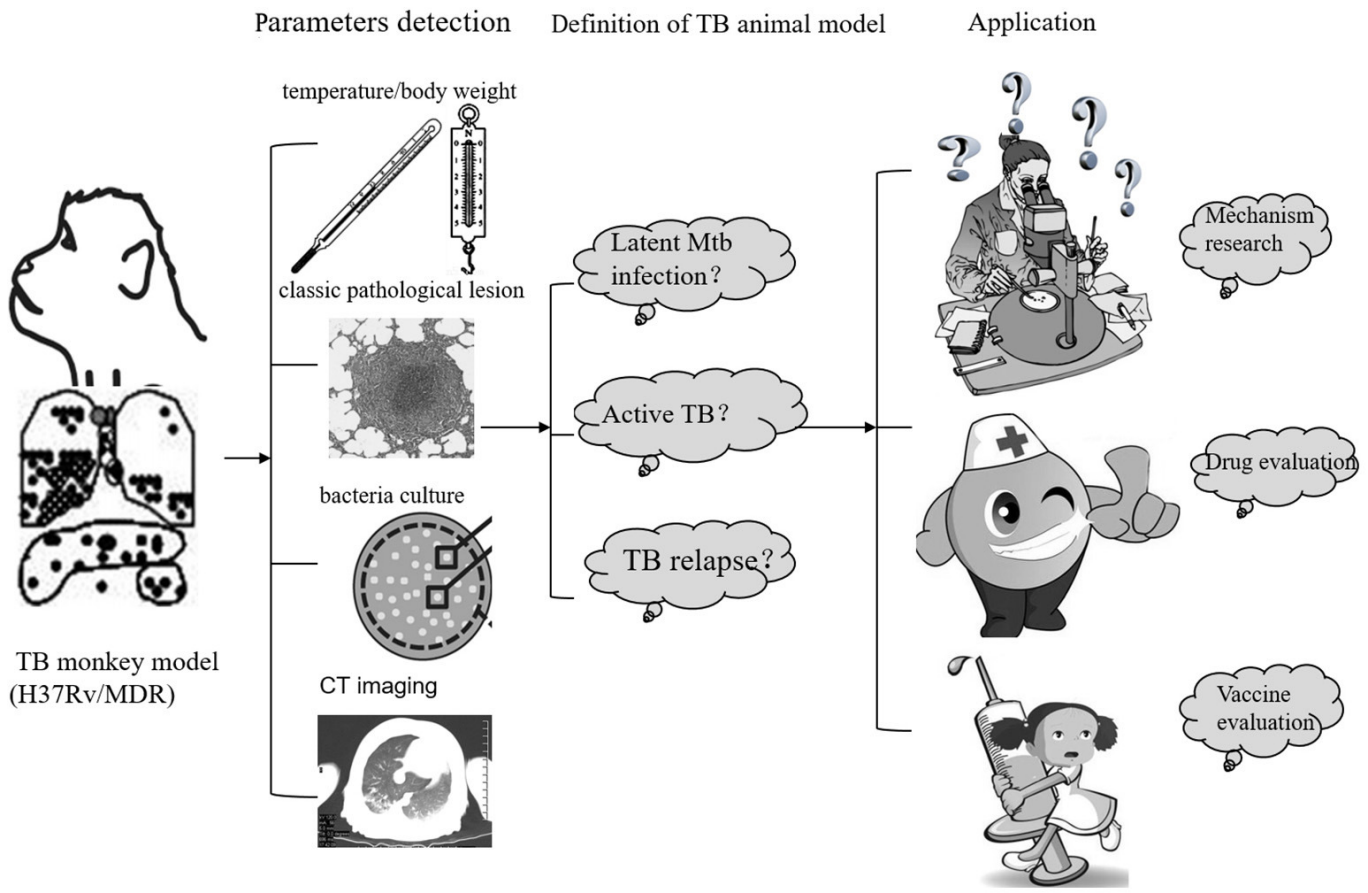
**The establish and application of monkey TB model**.

The current key problems of TB research refer to difficulty of MDR treatment, prevention of latent-relapse TB, treatment duration shorten, inefficiency of diagnosis, and treatment of extra-pulmonary tuberculosis (EPTB), complexity of TB and individual treatment, shortage of efficient TB vaccine and early diagnosis biomarkers. To investigate the above issues, lots of specific TB animal models are required. Vice versa, different TB animal model is used to research different issue (Figures 1, 2).

### Lack of specific biomarkers for early diagnosis of active tuberculosis

Although diagnosis, treatment, and prevention are equally important to halt the TB epidemic, accurate early diagnosis is the headstone of TB control (Abubakar et al., 2016). Currently, diagnosis of active TB is mainly based on sputum Mycobacterium tuberculosis (*Mtb*) culture, X-ray/Computed Tomography (CT) radiography, and tuberculin skin test (TST), along with clinical signs and patient history. The gold standard for TB diagnosis is sputum *Mtb* culture, which requires the presence of necrotic infection foci in proximity to the airways. This test also results in a 3–4-week delay before providing definitive results. Furthermore, current diagostic tests commonly require *Mtb*-positive sputum. However, many active TB patients, including HIV-TB co-infected individuals, those with extra-pulmonary TB, diabetes patients, and children, do not present with *Mtb*-positive sputum. Therefore, sputum *Mtb* culture is not very helpful for rapid and specific diagnosis of all TB types.

Active TB can also be diagnosed using other specimens or indexes, such as: *Mtb* DNA, *Mtb* Ag85 complex, or *Mtb* cell wall component lipoarabinomannan (LAM) in blood or urine; cytokines such as IP10\VEGF\HO-1 in serum; transcriptomic or metabolomic signatures; and phenotypes of PBMC detected by Fluorescence Activated Cell Sorting (FACS), Enzyme-Linked Immuno Sorbent Assay (ELISA), and Enzyme-Linked Immunospot Assay (ELISPOT). However, urine LAM detection has low sensitivity, blood and urine Ag85 show highly variable performance in different studies, and transcriptional profiles are complex and expensive to use as routine diagnostic tests, especially in TB epidemic areas (Goletti et al., 2016). A rapid detection method with high specificity but low cost is highly desirable, especially for TB epidemic areas where access to experimental facilities is usually very limited (Ray et al., 2013; Goletti et al., 2016).

Biomarkers are identified by comparison between latent TB infection and active TB in animal models. Among them, mouse and monkey model are the most commonly used TB models. In the monkey model, sputum or bronchial alveolar lavage fluid culture do not show definitive results and Erythrocyte Sedimentation Rate (ESR)>15 until 2 months post-infection, and the tuberculous granulomas are not seen before the 3rd week post-infection with positron emission tomography-computed tomography (PET-CT) (Ankrah et al., 2016). Immunologic changes cannot be generally detected until 3rd–4th week after infection in the mouse TB model.

It is urgent to establish a more stable active-latent-relapse TB model, which is essential for developing biomarkers for earlier and simpler detection of TB. Biomarkers for each stage of TB and for both *Mtb* and host are particularly needed and can be cytokines, chemokines, and/or immunologic cells in blood and urine (Phuah et al., 2016).

### Lack of efficient and reliable evaluation system for vaccine development

At present, BCG is the only vaccine approved for TB. Unfortunately, BCG is only effective in preventing tuberculous meningitis in children, not for adult TB (Ottenhoff and Kaufmann, 2012). Thus, it is necessary to develop new vaccines for TB. However, there is no efficient and reliable system to evaluate the efficacy of candidate vaccines. The new candidate vaccine MVA85A was once regarded as the most promising boosting vaccine after BCG vaccine since 1990s when tested in animal models, but the results of phase III clinical study were disappointing (Checkley and McShane, 2011; Ottenhoff and Kaufmann, 2012). The problem might be rooted in lack of efficient and reliable evaluation systems for long-term protective immunity. The desirable immunological biomarkers should be capable of predicting TB relapse, helpful in formulating immunizations, and speed up vaccine research and “bench to bed” translation (Thakur et al., 2012). Ideal TB animal models and evaluation systems are efficient and reliable tools to evaluate vaccines (Iwenofu et al., 2008), immune molecules, and cells as biomarkers in different TB stages and can thus be essential for early diagnosis and vaccine evaluation (Agranoff et al., 2006; Walzl et al., 2008; Zanini et al., 2008; Thakur et al., 2012).

### Side effects and duration of active TB treatment must be reduced

The standard chemotherapy for TB consists of 6 months of initial treatment and 8 months of retreatment. Treatment of drug-resistant TB types may last as long as 2 years (Stagg et al., 2016). It was reported that the duration of treatment could be reduced from 6 to 4 months with equal outcomes, animal models can be used to determine whether treatment time can be further shortened with better efficacy and fewer side effects (Nahid et al., 2016). For example, chemotherapeutic drugs can be evaluated using the acute active TB model (Swanson et al., 2016), while drugs such as immune agents that target the host can be evaluated using chronic or latent TB infection models. Pretreatment with Vitamin D or lucid ganoderma reveals no obvious anti-TB effect in active TB models but clear effects in latent TB animal models. The selection of animal models for drug evaluation depends on the type of drugs (Zhang et al., 2009; Gouveia et al., 2013; Swanson et al., 2016).

### Difficulty in multi-drug resistant (MDR) TB treatment

Treatment of MDR TB usually lasts 24 months, with 50% recovery worldwide, resulting in severe threatens to public health and economic losses (Stagg et al., 2016). It is necessary to employ methods of quick diagnosis and regimens of shorter and cheaper treatment to achieve fast diagnosis and improve disease prognosis (van Cutsem et al., 2016). Such methods and regimens can be developed and/or optimized based on MDR TB animal models. However, there is no active or latent infection MDR TB animal model in any species. It is imperative to establish MDR TB animal models (Harris et al., 2016; Stagg et al., 2016; van Cutsem et al., 2016; Zumla et al., 2016).

### Lack of systematic shorter and stable tuberculosis latent-relapse infection animal models

Research on early diagnosis and relapse prevention of latent tuberculosis infection (LTBI) in human populations is difficult but could be facilitated by using animal models, such as latent-relapse TB mouse, monkey, rat, or rabbit models (Kashino et al., 2008; Klinkenberg et al., 2008; Manabe et al., 2008; Elwood et al., 2009; Lin et al., 2009; Zhang et al., 2009; Singhal et al., 2011a; Subbian et al., 2012), among them, mice and monkey models are the most commonly used. The mouse model was established by spontaneous infection and intervened with anti-TB drugs and BCG. The defects of the mouse models include varied length of latent period, high bacterial burdens during the latent stage, and varied starting time-points and levels of relapse (Klinkenberg et al., 2008; Zhang et al., 2009; Shi et al., 2011; Murawski et al., 2014; Han et al., 2016; Kupz et al., 2016). The monkey LTBI models might be the second most common, but the LTBI in monkey can be defined only when the infection relapses; it cannot be predicted at the time of inoculation. Moreover, the latent phase lasts several years with big variation within groups (Lin et al., 2009; Pena and Ho, 2015). Thus, the existing models are not suitable for utility in research, and models with shorter and stable latent-relapse stages should be generated.

### Inefficient diagnosis and treatment of extra-pulmonary tuberculosis (EPTB)

Latent TB infection can progress to active TB, including EPTB, when the immunity of host decreases due to aging, stress, overuse of immunosuppressants, or co-infection with HIV. EPTB, in sites such as bone, lymph nodes, and meninges, often has high morbidity. Diagnosis and treatment of EPTB is difficult, posing a major threat to public health (Ray et al., 2013; Nahid et al., 2016). The clinical symptom of EPTB are usually obscure, so diagnosis is difficult. Sputum *Mtb* culture is not feasible in EPTB, and examination of tissues from lesion sites and liquid samples would help to make a definite diagnosis. Tissue examination of EPTB with biomarkers of *Mtb* and host is not easily implemented in patients; however it is feasible in EPTB animal models, which may facilitate development of definite diagnosis methods. It is urgent to develop simpler, cheaper, and specific diagnostic tools (Ray et al., 2013).

Standard chemotherapy has limited efficacy on EPTB. Surgery combined with standard chemotherapy is usually essential for bone and lymph node TB (Harris et al., 2016; Rahyussalim et al., 2016). Treatment of EPTB is difficult and treatment duration is always longer than that of standard chemotherapy (Reichardt, 2012; Jullien et al., 2016).

### Complexity of TB features

There are multiple TB types, including pulmonary and extra-pulmonary TB, LTBI, and various active TB formats. Research on the pathogenic mechanisms and evaluation of drugs and vaccines for a specific TB type must be based on animal models with individualized and precise target TB type (Peng et al., 2015; Abubakar et al., 2016; Kupz et al., 2016; Phuah et al., 2016; Zumla et al., 2016). Most TB features can be found in the TB monkey model, but results may vary within groups. TB monkey models can be used for research in precision medicine (Lin et al., 2009; Pena and Ho, 2015). Cross-collaborative (CC) mice or specific gene knockout mice TB models are the basic tools in TB's precision medicine research (Rogala et al., 2014; Elbahesh and Schughart, 2016).

## Progress in TB animal model preparation

An ideal TB animal model is one not only with *Mtb* infection but also from which viable *Mtb* can be isolated from tissues with standard parameters and which consistently mimics TB clinical signs, characteristic pathological lesions, bacterial loads in organs, immunological indexes, radiographic changes, and hematological changes (Shi et al., 2011; Waters et al., 2011; Subbian et al., 2012; Clark et al., 2015; Myllymaki et al., 2015; Pena and Ho, 2015; Kramnik and Beamer, 2016). A TB animal model can always be improved with classic disease characteristic, shorter disease progression and better practical utility. (Figure 2)

### Duration and biological safety of active TB animal models

Active TB models can be prepared in a variety of experimental animals for drug and vaccine evaluation, but it takes at least 2 months to complete *in vivo* research and evaluation (Gouveia et al., 2013; Izzo et al., 2015; Zhan et al., 2015; Conde et al., 2016; Swanson et al., 2016). This lengthy experimental time hinders the development of new drugs and vaccines. The main method for model evaluation comprises: detection of characteristic pathological lesions, determination of bacterial loads in target organs, observation of related clinical manifestations, tests for immunological changes in blood, and assessment of radiological changes. Although these methods are established and useful, they are time-consuming, inefficient, and have bio-safety risks. By increasing the dose of inoculation to induce more severe disease or by using more susceptible candidate strains, such as C3HeB/FeJ (Lanoix et al., 2015; Henao-Tamayo et al., 2015; Li et al., 2015), TNF-α or IFN-γ knockout mice (Ehlers et al., 2001; Manca et al., 2001; Turner and Orme, 2004; Green et al., 2013; Dorhoi et al., 2014; Francisco et al., 2015; Olleros et al., 2015), the onset of TB and evaluation process in mice could be accelerated above that of C57BL/6 or BALB/c. In addition, further applications of *in vivo* tracer techniques could facilitate detection of the abundance and distribution of the bacillus, as well as continuous monitoring of lesion progress in target organs; using these approaches could improve the accuracy of pre-clinical research, have higher detection sensitivity and safety, and optimize the use of laboratory animals (Sugawara et al., 2009; Kong and Cirillo, 2010; Kong et al., 2010, 2016; Zhang et al., 2010; Ozeki et al., 2015; Kato et al., 2016).

### The latent-relapse tuberculosis animal models

At present, preparation of mouse and monkey latency-recurrence infection models requires a long time and exhibits poor consistency in replication. Meanwhile, lack of biomarkers to predict long-term recurrence of TB remains a main disadvantage for evaluation of vaccine and drug candidates. Various immune agents and drugs have been used to achieve latency infection and relapse with variation among animals within group (Scanga et al., 1999). These models can be significantly improved by reducing the latency period and increasing the stability of the progress of disease latency and recurrence (Lenaerts et al., 2004; Nuermberger et al., 2004; Elwood et al., 2009; Lin et al., 2009; Shi et al., 2011; Subbian et al., 2012).

It is possible to acquire relatively shorter and more stable cycles of latency-relapse after anti-TB treatment or immunization using more susceptible strains such as C3HeB/FeJ, and TNF-α or IFN-γ-knockout mice (Turner and Orme, 2004; Calderon et al., 2013; Henao-Tamayo et al., 2015; Olleros et al., 2015; Reeme and Robinson, 2016) or other sensitive strains screened from collaborative cross (CC) mice (Rogala et al., 2014; Elbahesh and Schughart, 2016). On the other hand, rapid and uniform relapse with moderate bacterial loads and lesion severity in target organs can be attained using certain hormones, new specific immunosuppressants such as TNF-α antibodies, or gamma irradiation; this approach could contribute to the statistical analysis in experiment due to the little variation within group (Scanga et al., 1999; Botha and Ryffel, 2002; Parikka et al., 2012; Goletti et al., 2016). Imaging detection can also help to shorten the latency course monitoring and allow earlier determination of the relapse phase (Botha and Ryffel, 2002; Zhang et al., 2010, 2012; Murawski et al., 2014; Kramnik and Beamer, 2016).

### Drug-resistant (DR) TB animal models

A qualified drug-resistant animal model for efficacious evaluation of new drugs or drug combinations should possess common resistance phenotypes, typical drug-resistant mutations, and representative genotype background DR *Mtb* strains (van Cutsem et al., 2016). Strong virulent strains with these features could be applied in rapid assessment of drug efficacy, and moderate virulent strains could be used in the research of immune agents and immunological mechanism (HaiRong et al., 2014). Unfortunately, although we screened standardized drug-resistant strains to inoculate animal models, there remain no specialized drug-resistant latent and active TB models or evaluation system reported (D'Ambrosio et al., 2015; Olmo et al., 2016) and no latency-relapse animal drug-resistant TB model reported till now, either.

### Monkey tuberculosis models

Notably, monkey TB models exhibit inter-individual differences, probably due to genetic variation in their populations. This is an advantage in terms of similarity to human population but a challenge in terms of uniform responses. These characteristics are similar for MDR TB in monkeys. The disadvantage might be reduced by improving the inoculation dose\the *Mtb* strain and application of intervention (Scanga and Flynn, 2014; Pena and Ho, 2015; Phuah et al., 2016). For preparation of an active tuberculosis model, inoculation dose could be increased to make disease progression more consistent (Zhang et al., 2014). This is in contrast to preparation of latent infection mouse or guinea pig models, where inoculation can be optimized with moderate doses and intervention with drugs or immunosuppressive agents (Lenaerts et al., 2004; Nuermberger et al., 2004; Lu et al., 2015, 2016) can improve the stability of latent status and reduce deviation of recurrence. In addition, to replicate primary syndrome and single granulomatous lesions in monkeys, local inoculation of moderate virulent strains should be tried in small doses (Cluver et al., 2013).

### *In vivo* monitoring of animal TB models

At present, TB animal models can be continuously evaluated through sampling sacrificed animals at multiple time points. This requires a huge number of experimental animals and increases bio-safety risks. There is also inter-individual variation within experimental groups (Zhan et al., 2015). These problems can be solved by *in vivo* monitoring technology. 18F-FDG PET/CT was commonly used in early diagnosis of infectious inflammation. FDG imaging was highly dependent on the strength of immune response by host cells; thus, the image was quite blurry in immunity-compromised hosts. Technologies such as FDG PET/CT should be improved to be sensitive enough for detection of pathogen, including bacterial localization and quantification (Murawski et al., 2014; Weinstein et al., 2014; Kato et al., 2016). FDS PET/CT is also a promising method for *in vivo* detection of bacterial infection (Weinstein et al., 2014; Ordonez et al., 2016; Yao et al., 2016). Fluorescence-labeled (GFP, reporter enzyme fluorescence) bacillus could be detected *in vivo*, including the location and quantity of the pathogen. However, the current detection sensitivity is above 100 CFU, which is not sensitive enough to diagnose latent infection. The structure or features of fluorescent proteins should be modified to achieve a lower *in vivo* detection limit (Sugawara et al., 2009; Kong et al., 2010; Kong and Cirillo, 2010; Ozeki et al., 2015).

## Perspectives on animal TB models

The difficulty of research with TB animal models is that the pathogenesis and progression of TB are complex (Ottenhoff, 2012). Infection type can vary by lesion location, with manifestations as both pulmonary and extra-pulmonary (e.g., bone, lymphatic, enterophthisis, meningeal) TB (Myllymaki et al., 2015). The disease progression has diversified forms: latent and active infection. Active TB includes primary tuberculosis, blood disseminated tuberculosis, and secondary tuberculosis (Lin et al., 2009; Waters et al., 2011; Subbian et al., 2012; Clark et al., 2015; Kramnik and Beamer, 2016). A single TB animal model can only mimic one or several aspects of TB, not all forms. To reveal the overall picture of human TB, all features of TB should be replicated in various TB models for different research purposes (Rodgers et al., 2007; Kashino et al., 2008; Manabe et al., 2008; Dannenberg, 2009; Zhang et al., 2009; Waters et al., 2011; Parikka et al., 2012; Subbian et al., 2012; Baldwin and Telfer, 2015; Rahyussalim et al., 2016; Swanson et al., 2016).

### Animal TB models for rapid drug and vaccine assessment

It is urgent to develop animal models with rapidly-progressing TB for drug and vaccine evaluation. It may be faster to evaluate drug efficacy for survival using IFN-γ or TNF-α knockout active TB mice than wild-type TB mice. TNF-α KO mice might be useful in preparation of a latency-recurrence TB model for evaluation of drugs and vaccines. After 8 weeks of anti-tuberculosis chemotherapy, TNF-α KO mice can experience natural recurrence and death (Turner and Orme, 2004; Francisco et al., 2015; Olleros et al., 2015). Relapse can be induced in monkeys with TNF-α antibody, generating a homogenous rapid latent-relapse TB monkey model (Lin et al., 2010; Fillmore et al., 2012).

### Application of new detecting technologies on animal TB models

New technology should be exploited to improve sensitivity and specificity of detection, shorten the course of experiments, decrease systematic error by monitoring the same individuals continuously instead of sampling different dissected animals at different time points, increase reliability, and reduce bio-safety risk for personnel. For example, bacteria imaging and PET-CT were promising methods for bacillus and lesions imaging (Weinstein et al., 2014; Ankrah et al., 2016; Lin et al., 2016; Ordonez et al., 2016).

### Application of new gene-modified technologies on animal TB models

Specific gene-editing technology, including gene knock-in, knock-out, and knock-down with CRISPR-Cas9 or other new techniques, has been applied in mice, rats, and even monkeys in research of pathogenesis. This approach has enabled interpretation of precise roles of genes and proteins (Commandeur et al., 2014; Gengenbacher et al., 2014; Armstrong et al., 2016; Cheong et al., 2016; Jung et al., 2016; Lambert et al., 2016; Ma et al., 2016; Renaud et al., 2016) and elaboration of mechanisms for treatment and vaccine strategies.

### Personalized animal TB models

Multiple TB animal models should be established for specific purposes. For example, different immunization models are needed, for prime immunization, prime-boost immunization, and therapeutic vaccine evaluation. These models have been established in guinea pigs (Lu et al., 2015, 2016). A gene-knockout mouse TB model with virulent strains can be used for fast evaluation of chemical drug efficacy (Ehlers et al., 2001), while standard and attenuated strains can be used in evaluation of *in vivo* immune responses and phenotype of immune agents. A monkey model is suitable for personalized medical research by *in vivo* monitoring methodologies and can be used in systematic research of early diagnosis and treatment of TB recurrence (Capuano et al., 2003).

### Extra-pulmonary TB models

Models for extra-pulmonary TB are especially useful in research on pathogenesis and treatment. New technologies such as FDG-PET/CT and fluorescent-labeled imaging can also be used in EPTB models to improve sensitivity of diagnosis and rapid evaluation of drug efficacy, such that new biomarkers for diagnosis and evaluation systems can be explored.

## Summary

TB animal models have been playing essential roles in the translational and precision medicine, not only in areas of early diagnosis, recurrence prevention, treatment optimization of MDR-TB, and EPTB, with the thinking of personalized medicine, but also for the new vaccine and drug development. Each TB animal model has its own advantages and disadvantages, thus lots of specific TB animal models are required for the above research. It is imperative to improve existing TB animal models to obtain more stable, more accurate, and more efficient models with shorter experimental cycles and lower bio-safety risks using new technologies and strategies.

## Author contributions

CQ and LZ conceived the idea, LZ wrote the manuscript, JT and MS revised the manuscript.

### Conflict of interest statement

The authors declare that the research was conducted in the absence of any commercial or financial relationships that could be construed as a potential conflict of interest.
